# Improved language function for post-stroke aphasia in the long term following repeated repetitive transcranial magnetic stimulation and intensive speech-language-hearing therapy: a case report

**DOI:** 10.1186/s13256-023-03992-0

**Published:** 2023-07-08

**Authors:** Yoshihiro Sakurai, Masahiro Abo, Saki Terajima, Saho Ikeda, Kensuke Ohara, Takuya Hada

**Affiliations:** Department of Rehabilitation Medicine, The Jikei University School of Medicine, 3-25-8, Nishi-Shimbashi, Minato-ward, Tokyo, 105-8461 Japan

**Keywords:** Stroke, Aphasia, Rehabilitation, Repetitive transcranial magnetic stimulation, Intensive speech-language-hearing therapy

## Abstract

**Background:**

There have been no papers reporting improvement in language function and changes in cerebral blood flow following repeated use of repetitive transcranial magnetic stimulation in combination with intensive speech-language-hearing therapy. This case report concerns the efficacy of repeated use of repetitive transcranial magnetic stimulation and intensive speech-language-hearing therapy for a certain patient suffering from aphasia following stroke, plus the findings of the cerebral blood flow measurements.

**Case presentation:**

The patient was a 71-year-old right-handed Japanese male who developed fluent aphasia following a left middle cerebral artery stroke. He underwent repetitive transcranial magnetic stimulation and intensive speech-language-hearing therapy five times in total. The repetitive transcranial magnetic stimulation was applied to right inferior frontal gyrus at the frequency of 1 Hz plus 2 hours per day of intensive speech-language-hearing therapy. The patient’s language function was evaluated in the short term and long term. Cerebral blood flow was measured with single photon emission computed tomography scan. As a result, in the short term, the patient’s language function improved especially during the initial hospitalization. In the long term, it improved gradually and stabilized.Cerebral blood flow was increased in the right hemisphere.

**Conclusions:**

The findings of the study indicate that the repeated use of repetitive transcranial magnetic stimulation and intensive speech-language-hearing therapy may be effective in improving and preserving language function and increasing cerebral blood flow for aphasia following stroke.

## Background

Around 21% to 40% of stroke patients sustain permanent aphasia [1]. Stroke survivors with acquired language disability are generally thought to reach a plateau within a year of stroke onset, after which their residual language abilities stabilize [2]. Treatment with repetitive transcranial magnetic stimulation (rTMS) for post-stroke aphasia facilitates recovery of language function by regulating cerebral cortex excitability. The department of rehabilitation medicine of The Jikei University School of Medicine started performing rTMS therapy for patients with aphasia following stroke in 2008, and reported the efficacy of the therapy [3–10]. The latest international rTMS guidelines 2020 recommend rTMS therapy in post-stroke aphasia as level B of evidence (probable efficacy) [11].

There have been no reports in which the repeated use of rTMS in combination with intensive speech-language-hearing therapy (iST) was performed in patients with post-stroke aphasia together with evaluation of the treatment course by cerebral blood flow (CBF) measurements. We performed rTMS and iST as an intensive rehabilitation treatment five times for a patient with post-stroke aphasia, by which long-term language function recovered. The findings, as well as cerebral blood flow measurements by single photon emission computed tomography (SPECT) scan, are reported herein.

## Case presentation

The patient was a 71-year-old Japanese male who was naturally right-handed, with no history of being forced to use his right hand. He was a university graduate and was an executive officer of a company. His past medical history included atrial fibrillation, hypertension, and diabetes mellitus. Having developed left middle cerebral artery stroke, the patient was treated conservatively because thrombus retrieval therapy and thrombolysis were not yet available in Japan at that time. Seven days after onset, when his condition had stabilized, he started speech-language-hearing therapy (ST). Two months after onset, the patient was discharged from hospital and continued ST on an outpatient basis. However, there was little improvement in speech and language function, so he visited the department of rehabilitation medicine at The Jikei University School of Medicine at 9 months after stroke onset.

**Fig. 1:**
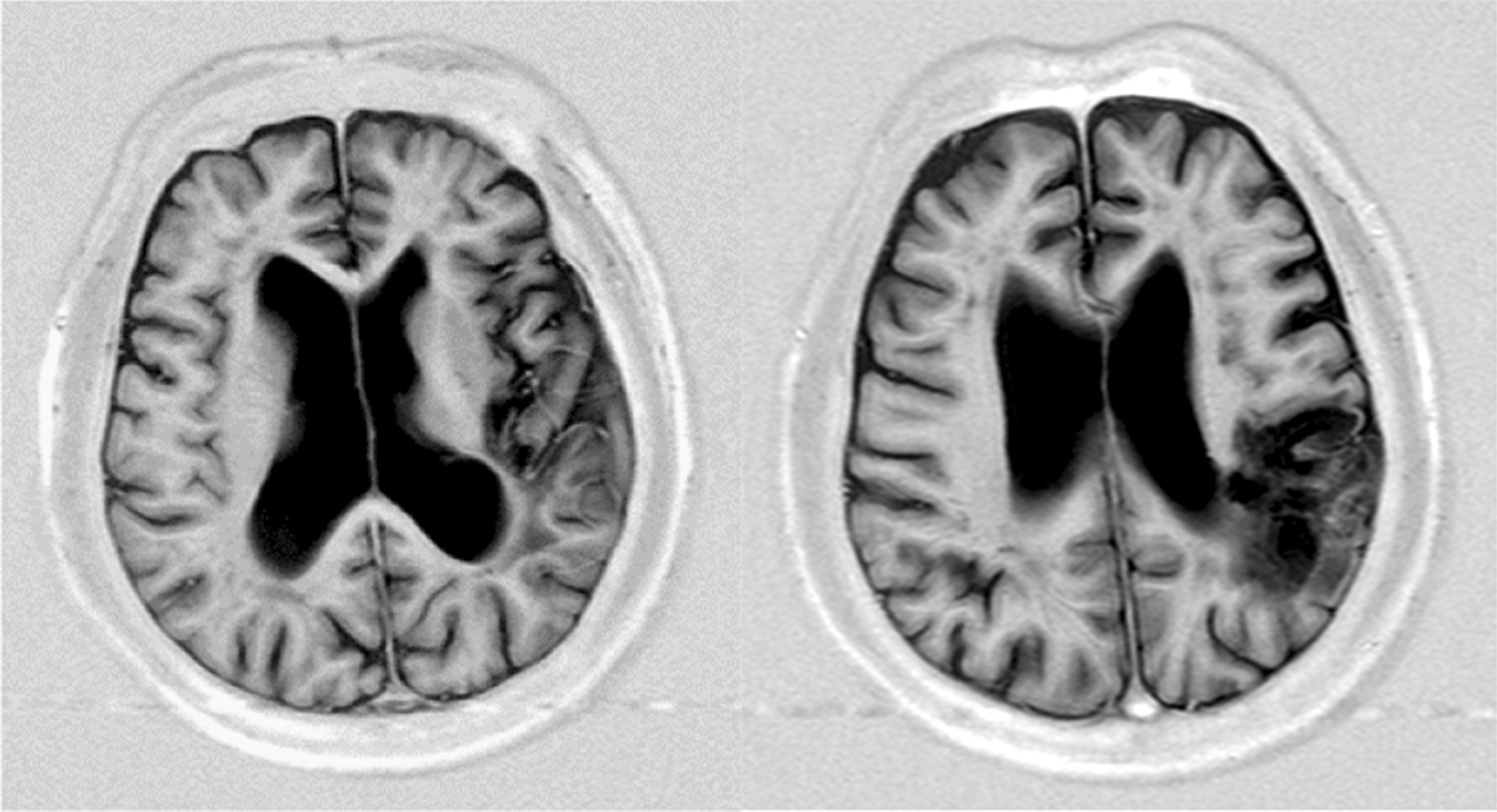
MRI T1 weighted image of the patient’s head. T1-weighted magnetic resonance imaging (MRI) of the head showed low signal regions from the left temporal lobe to supramarginal gyrus and inferior parietal lobule. *MRI*: Magnetic resonance imaging

The findings at the initial visit were as follows: the patient exhibited good awareness with no paralysis in gross motor function. The cognitive function score by functional independence measurement (FIM) was 31/35 points. The scores were 6, 4, 7, 7, and 7 in the order of communication (comprehension, expression) and social cognition (social interaction, problem solving, and memory), respectively. The brain imaging test using T1-weighted magnetic resonance imaging (MRI) of the head showed low signal regions from the left temporal lobe to supramarginal gyrus and inferior parietal lobule (Fig. 1). The Standard Language Test of Aphasia (SLTA) results showed a greater language loss function in terms of ability to speak and write compared with aural and reading comprehension. His utterance was fluent in short sentences. No anarthria was observed, but phonemic paraphasia and neologism were observed for verbal expression. He had phonological spelling errors in writing. The neuropsychological test findings showed that the Raven’s Colored Progressive Matrices (RCPM) score was 26/36 points (the average in the 70s being 26.9 points).

He underwent inpatient intensive rehabilitation treatment consisting of rTMS and intensive ST (iST) five times in total (at 10 months, 20 months, 27 months, 35 months, and 47 months after stroke onset). Each duration of hospital stay was 13 days. He continued to undergo ST periodically on an outpatient basis. Table 1 shows therapeutic regimens during hospitalization.

**Table 1:** Patient schedule for 2-week rTMS in combination with iST hospitalization

	Monday	Tuesday–Saturday	Sunday	Monday–Friday	Saturday
AM	Admission	rTMS	None	rTMS	rTMS
		Individual ST		Individual ST	Individual ST
	Lunch break			Lunch break	
PM	Evaluation at admission	Individual ST		Individual ST	Evaluation at discharge
	rTMS	Voluntary training		Voluntary training	Discharge
	Individual ST				

Language function was evaluated on admission and discharge. ST was performed for a total 2 h per day. Patients received rTMS at 1 Hz for 2400 pulses per day for a total of 14,400 pulses per week

*rTMS* repetitive transcranial magnetic stimulation, *ST* speech-language-hearing therapy

### Repetitive transcranial magnetic stimulation (rTMS)

The rTMS was performed using a eight-shaped coil with a diameter of 70 mm and MagPro R30 Stimulator (MagVenture Company, Denmark). The location of rTMS stimulation was right inferior frontal gyrus (IFG) [6, 12]. The motor threshold was measured at which movements of the first dorsal interosseous muscle of the left upper limb were visually observed when the right primary motor cortex was stimulated. The stimulation intensity was set at 90% of the motor threshold. The stimulation frequency was set at a low frequency of 1 Hz. The stimulation time was 40 minutes per session. A total of 2400 pulses of rTMS were applied per day. He underwent rTMS session once a day with a total of 12 sessions, excluding Sunday, during hospitalization.

### Intensive speech-language-hearing therapy (iST)

He underwent iST with a total of 120 minutes per day during hospitalization. One-on-one ST training with the intent of improving phonological awareness was performed by a speech-language-hearing therapist. The main training included reading aloud short sentences written only in the Japanese syllabary characters and phonological manipulation tasks (phonological extraction, mora counting, written naming, dictation, and adding Japanese syllabary characters by providing the framework of the mora). He encouraged voluntary training in the wards.Upon discharge from the hospital, he and his family received instruction about communication methods from the speech-language-hearing therapist.

### Evaluation of language function

The patient’s language function was evaluated by the Standard Language Test of Aphasia (SLTA), the Supplementary Tests for Standard Language Test of Aphasia (SLTA-ST), the Japanese version of Western Aphasia Battery (J-WAB), and the Japanese version of Token Test (J-Token Test). The speech-language-hearing therapist scored each test according to the prescribed scoring method. The patient underwent the naming task of the SLTA-ST, the shadowing task of the J-WAB and the J-Token Test as the short-term evaluation for language functions at both the time of hospitalization and hospital discharge 2 weeks later. He also received the SLTA as long-term assessment for language functions during hospitalization and at 3 months after hospital discharge.

The SLTA is widely used as the standard language test for people who are native speakers of Japanese [6, 13]. The SLTA is designed to evaluate language functions including utterance, naming, shadowing, auditory comprehension, reading comprehension, writing, and calculation. Four domains of the SLTA (listening, speaking, reading, and writing) were assessed. The scores of SLTA subtests were converted into *Z*-scores by utilizing the mean and standard deviation. The mean *Z*-score of listening, speaking, reading, and writing of the SLTA by each domain, as well as the mean of *Z*-scores of the SLTA four domains were calculated.

The SLTA-ST consists of more complicated tasks than SLTA and is designed to detect symptoms of mild language disability, which the SLTA cannot [5]. The naming task of the SLTA-ST was performed using 80 words. The J-WAB is designed to measure linguistic skills and auditory comprehension. The shadowing task of the J-WAB was performed with 100 points as the maximum score. The J-Token Test is used to detect slight language comprehension deficits; severity of aphasia correlates to the test score. The maximum score of the J-Token Test was 165 points.

### Single photon emission-computed tomography (SPECT) and laterality index (LI)

The patient’s regional cerebral blood flow (rCBF) was measured with SPECT scan. 99mTc-ethyl cysteine dimer (99mTc-ECD) was used as a tracer. The SPECT scan was performed three times in total (at 20 months, 30 months, and 47 months after stroke onset). The patient was injected intravenously with 99mTc-ECD (600 MBq) and the SPECT scan was performed 20 minutes later. The conditions at SPCET scanning included eyes closed resting states in the decubitus position. The SPECT imaging software used for quantitative analysis included three-dimensional stereotactic regions of interest template (3DSRT) and fine stereotactic regions of interest template (FineSRT).

The 3DSRT and FineSRT are designed to quantitatively calculate CBF in the region of interest (ROI). The rCBF was set at the mean CBF of each ROI (mL/100 g/minute) [19]. A difference of CBF in right and left cerebral hemispheres was calculated using rCBF of language-related ROI as laterality index (LI) as follows [7]:

$${\text{LI }} = \, [({\text{right rCBF}} - {\text{left rCBF}}) \, / \, \left( {{\text{right rCBF }} + {\text{ left rCBF}}} \right)]$$

CBF in the affected side of the left cerebral hemisphere decreased. This means the higher LI value is, the more CBF in the right cerebral hemisphere increases compared with the CBF in the left hemisphere.

### Results of language function and cerebral blood flow

**Fig. 2:**
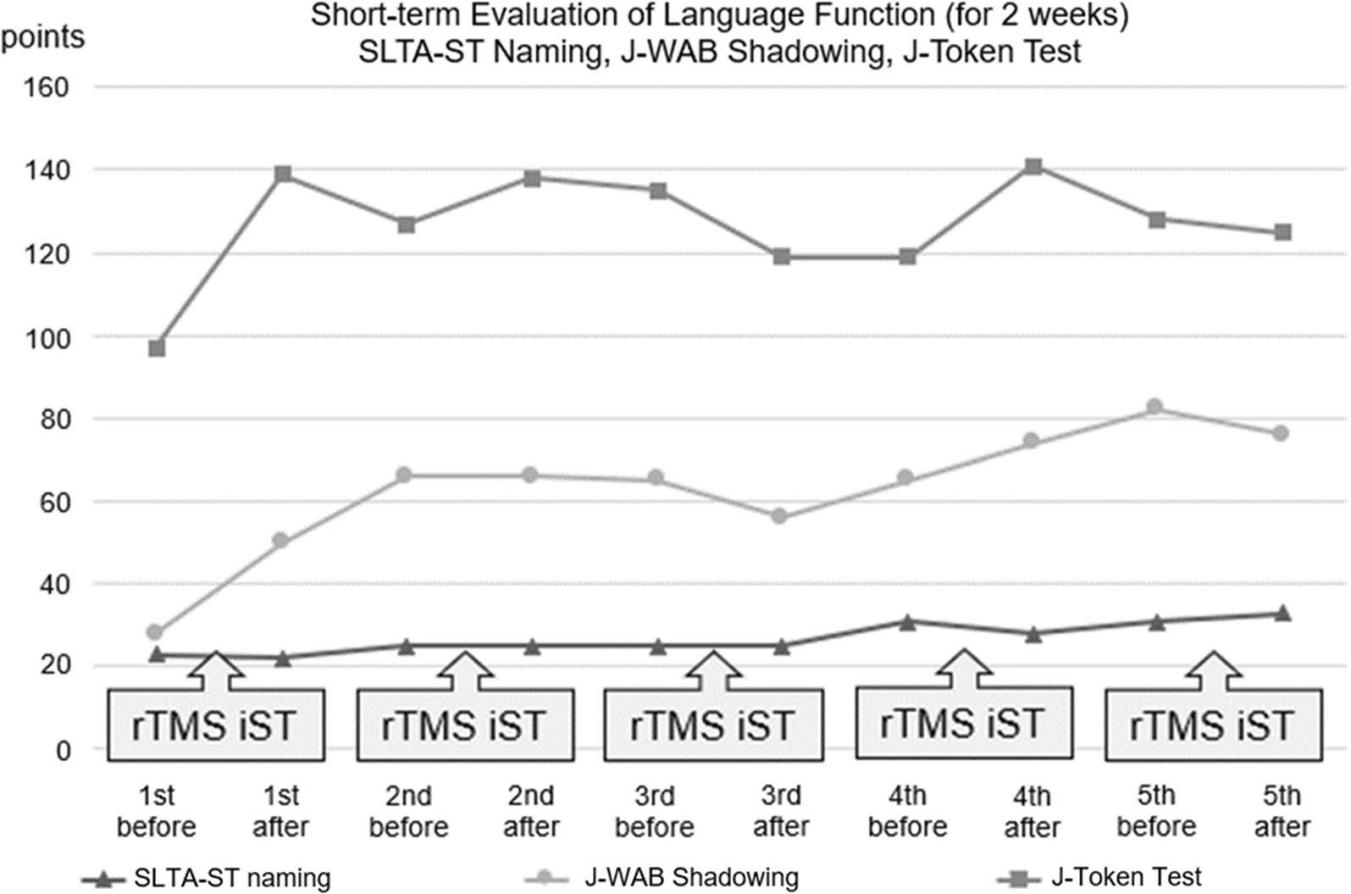
Evaluation of language function before and after 2-weeks repetitive transcranial magnetic stimulation in combination with intensive speech-language-hearing therapy. Transitions in the short-term evaluation during hospitalization (naming task of the Supplementary Tests for Standard Language Test of Aphasia, shadowing task of the Japanese version of Western Aphasia Battery and the Japanese version of Token Test) are shown. Improvement in short-term language function during hospitalization was remarkable in the initial hospitalization and stabilized after repeated hospitalization. Evaluation of language function is shown during hospitalization and 3 months after discharge. *SLTA-ST*: Supplementary Tests for Standard Language Test of Aphasia. *J-WAB*: Japanese version of Western Aphasia Battery. *J-Token Test*: Japanese version of Token Test. *rTMS*: Repetitive transcranial magnetic stimulation.* iST*: intensive speech-language-hearing therapy

**Fig. 3:**
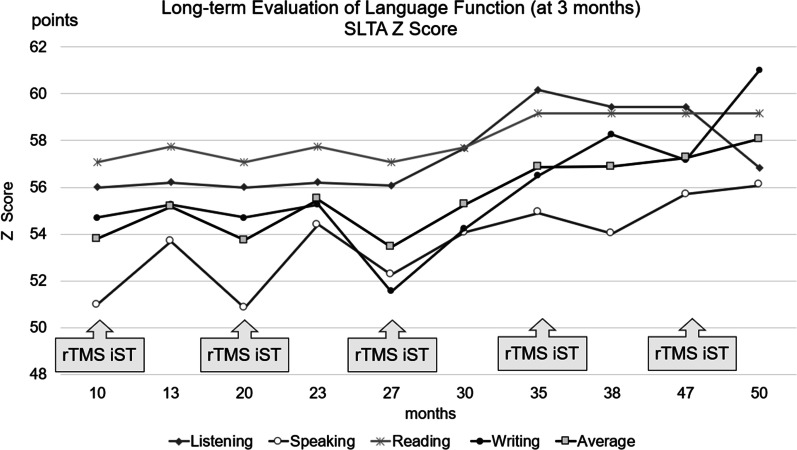
The transitions in the long-term language function evaluation (*Z*-score of the Standard Language Test of Aphasia) are shown for 3 months after hospital discharge. The *Z*-score of each Standard Language Test of Aphasia domain and the mean *Z*-scores of the Standard Language Test of Aphasia four domains improved simultaneously. The patient’s speaking and writing improved much more compared with listening and reading comprehension. *SLTA*: Standard Language Test of Aphasia.* rTMS*: repetitive transcranial magnetic stimulation.* iST*: intensive speech-language-hearing therapy.

The patient completed treatment without adverse events. Language function gradually improved over the long term, was maintained, and then stabilized. His speech improved from word level to short sentences, and the frequency of phonemic paraphasia decreased. Table 2 and Fig. 2 show transitions in the short-term evaluation during hospitalization (naming task of the SLTA-ST, shadowing task of the J-WAB and the J-Token Test). His scores for J-WAB improved from 28 to 50 points, and from 97 to 137 points for the J-Token Test during the initial hospitalization. Improvement in short-term language function during hospitalization was remarkable in the initial hospitalization and stabilized after repeated hospitalization. Table 3 and Fig. 3 show the transitions in the long-term language function evaluation (*Z*-score of the SLTA) for 3 months after hospital discharge. The mean *Z*-scores of four domains, listening, speaking, reading, and writing, improved from 53.8 to 58.1 points over the course of a long period of time from 10 to 51 months after the onset of stroke. The *Z*-score of each SLTA domain and the mean *Z*-scores of the four SLTA domains improved simultaneously. The patient’s speaking and writing improved much more compared with listening and reading comprehension.

**Table 2:** Evaluation of language function before and after 2-weeks rTMS in combination with iST

Short-term evaluation of language function (for 2 weeks)										
	1st rTMS and iST		2nd TMS and iST		3rd TM and iST		4th TMS and iST		5th TMS and iST	
	Before	After	Before	After	Before	After	Before	After	Before	After
SLTA-ST naming (80 points in total)	23	22	25	25	25	25	31	28	31	33
J-WAB shadowing (100 points in total)	28	50	66	66	65	56	65	74	82	76
J-Token Test (165 points in total)	97	139	127	138	135	119	119	141	128	125

As evaluation of language function before and after 2-weeks rTMS and iST, the naming task of the Supplementary Tests for Standard Language Test of Aphasia (SLTA-ST), the shadowing task of the Japanese version of Western Aphasia Battery (J-WAB), and the the Japanese version of Token Test (J-Token Test) were performed at each hospitalization

**Table 3:** Evaluation of language function during hospitalization and 3 months after discharge

Long-term evaluation of language function (at 3 months) of SLTA *Z*-score										
Months	10	14	20	24	27	30	35	39	47	51
Listening	56.0	56.2	56.0	56.2	56.1	57.7	60.2	59.5	59.5	56.8
Speaking	51.0	53.7	50.8	54.4	52.3	54.1	54.9	54.0	55.7	56.1
Reading	57.1	57.8	57.1	57.8	57.1	57.7	59.2	59.2	59.2	59.2
Writing	54.7	55.3	54.7	55.3	51.5	54.2	56.5	58.2	57.2	61.0
Average	53.8	55.2	53.7	55.5	53.4	55.3	56.9	56.9	57.3	58.1

Language function was evaluated during rTMS and iST hospitalization and 3 months after discharge; Standard Language Test of Aphasia (SLTA) was used as long-term evaluation. “Listening,” “Speaking,” “Reading,” and “Writing” subtests were administered and scores were *Z*-scored. *Z*-score averages for the four SLTA regions were calculated

LI increased, with CBF in right hemisphere increasing more than that of the left hemisphere. Table 4 shows transitions of rCBF in the parietal lobe, angular gyrus, and temporal lobe, as well as LI transitions using 3DSRT of the SPECT. Table 5 shows transitions of rCBF and LI in each language-related area using FineSRT of the SPECT.

**Table 4:** Regional cerebral blood flow (rCBF) and laterality index (LI) in 3DSRT analysis

rCBF in 3DSRT							
	20		30		47		Months
	Right	Left	Right	Left	Right	Left	
Parietal	34.48	23.65	34.67	22.05	39.30	23.15	
Angular	37.40	33.56	38.14	33.27	42.68	34.83	
Temporal	35.11	30.11	36.27	30.62	42.13	33.49	mL/100 g/min

LI in 3DSRT				
	20	30	47	Months
Parietal	0.1863	0.2225	0.2586	
Angular	0.0541	0.0682	0.1013	
Temporal	0.0767	0.0845	0.1143	

SPECT images were quantitatively analyzed using three-dimensional stereotactic regions of interest template (3DSRT). The regions of interest (ROI) were defined for each language-related region, and the averaged regional cerebral blood flow (rCBF) is shown

**Table 5:** Regional cerebral blood flow (rCBF) and laterality index (LI) in fineSRT analysis

rCBF in fineSRT							
	20		30			47	Months
	Right	Left	Right	Left	Right	Left	
Inferior frontal gyrus	37.84	34.23	38.74	34.10	45.56	37.50	
Superior parietal lobule	31.41	30.39	31.12	29.61	34.91	33.09	
Inferior parietal lobule	36.49	19.89	37.06	17.67	41.91	17.56	
Angular gyrus	37.52	33.44	38.25	33.16	42.81	34.66	
Supramarginal gyrus	34.57	19.23	35.64	17.96	42.12	16.68	
Superior temporal gyrus	34.59	28.01	35.54	28.47	42.60	31.80	
Broca	38.47	34.57	38.14	34.29	45.03	36.69	
Wernicke	37.68	29.83	38.69	29.53	45.65	31.69	
Insula	40.46	32.53	42.00	32.21	47.59	36.55	mL/100 g/minute

LI in 3DSRT				
	20	30	47	Months
Inferior frontal gyrus	0.0501	0.0637	0.0970	
Superior parietal lobule	0.0165	0.0249	0.0268	
Inferior parietal lobule	0.2944	0.3543	0.4095	
Angular gyrus	0.0575	0.0713	0.1052	
Supramarginal gyrus	0.2851	0.3299	0.4327	
Superior temporal gyrus	0.1051	0.1105	0.1452	
Broca	0.0534	0.0532	0.1021	
Wernicke	0.1163	0.1343	0.1805	
Insula	0.1086	0.1319	0.1312	

SPECT images were quantitatively analyzed with fine stereotactic regions of interest template (FineSRT). The regions of interest (ROI) were defined for each language-related region, and the averaged regional cerebral blood flow (rCBF) is shown

## Discussion and conclusions

### Discussion

The patient underwent intensive rehabilitation treatment consisting of rTMS in combination with iST several times, by which his language function improved. In short-term evaluation, his language function was markedly improved initially and stabilized after repeated hospitalization. In long-term evaluation, language function was initially improved in a stepwise manner, and after repeated hospitalization, it improved in a linear fashion and then stabilized. In a case report where intensive treatment with rTMS and iST was repeated four times, a stepwise improvement in the language function was noted. One study also reports that for rTMS in patients with non-fluent aphasia following stroke, low-frequency stimulation had a longer lasting effect on the language function than high-frequency stimulation [14]. It was furthermore found that short-term intensive ST improves language function in patients with aphasia in the chronic stage, after which it stabilizes it for an extended period of time [15]. Language function of our patient improved remarkably in the early stage, along with inpatient intensive rehabilitation therapy using rTMS in combination with iST; repeated intensive rehabilitation contributed to maintaining the improved language function for an extended period of time as well. Although an instant evaluation shows the language function reaches a plateau in the short term by repeated intensive rTMS and iST, the combination use of rTMS and iST has a long-term stabilizing effect on the language function. Thus, rTMS and iST should be continued if language function reaches a plateau in a short time.

For our patient, low-frequency rTMS was applied to right IFG based on international rTMS guidelines at that time [16]. The latest international rTMS guidelines 2020 recommend low-frequency rTMS to right IFG in post-stroke non-fluent aphasia [11]. This guideline, however, does not provide a consensus concerning the location of rTMS stimulation in patients with fluent aphasia. Regarding stimulation frequency, 5 Hz or higher-frequency rTMS releases excitatory neurotransmitters, whereas 1 Hz or lower frequency rTMS releases inhibitory neurotransmitters. Thus, high-frequency rTMS upregulates neuronal activity, low-frequency rTMS downregulates it [17]. At present, it is unknown whether the right or left cerebral hemisphere is significant in terms of the recovery course of language functions in aphasia in the chronic stage [6, 10].

In patients with post-stroke aphasia, the activation of right IFG may contribute to the compensation for loss of language function and its reacquisition. On the other hand, a study reports that the compensatory ability of the right IFG was restricted in terms of the improvement of language function compared with the group with the function activated in the left IFG [18]. Thus, when low-frequency rTMS with inhibitory neuronal activity is applied to right cerebral hemisphere, it may impair language function compensated by activating right cerebral hemisphere in some cases, and may facilitate the activation of original language function of left cerebral hemisphere in other cases. In our patient, an exacerbation of language function was observed only for the instant evaluation at the third hospitalization and hospital discharge before and after low-frequency rTMS to right IFG. The short-term and long-term evaluation during the other four times of hospitalizations showed a favorable improvement in his language function. Our patient indicates that low-frequency rTMS to the right IFG may be effective, not only in patients with post-stroke non-fluent aphasia, but also in those with post-stroke fluent aphasia.

In this case, low-frequency rTMS was applied to right IFG. When comparing the CBF in right and left cerebral hemispheres, an increased flow was observed in the right hemisphere. LI changes of rCBF are used for research on regulation and plasticity of neural circuit to evaluate the impact of rTMS on CBF [7]. The CBF was measured with SPECT scan by accumulation of radioisotope in the language-related area. rTMS for the treatment of post-stroke aphasia when used in combination with rehabilitation therapy promotes an improvement of language function. This paper reports research result on the effect of rTMS based on functional brain imaging [9]. However, even if an increased flow in the right cerebral hemisphere is observed, it does not necessarily mean that it upregulates the function in the right hemisphere, for which caution must be taken. In short, there is a possibility that neuronal functions may be suppressed by increasing the CBF in the stimulation area, as well as releasing inhibitory neurotransmitters.

After the treatment with low-frequency rTMS was performed for our patient, the CBF in the stimulation site increased and his speaking and writing ability especially improved. For the relationship between the location of cerebral hemispheres and language function, left IFG is associated with language generation, and the posterior region is especially involved in phonological processing [19]. Also, the insular cortex mediates motor aspects of speech production. According to a study, functional MRI (fMRI) findings showed that right and left anterior insular cortex, which is involved in speech production, was activated in healthy people and that the middle portion of insula in the left hemisphere involved in speech comprehension was activated [20]. While improved utterance was observed in our patient, a decrease in the approaching behaviors and phonemic paraphasia was observed, which was likely to be derived from the upregulated neuronal function in left IFG and left insular cortex, leading to the increase in the CBF in right IFG or right insular cortex on the SPECT images. The detailed mechanism of treatment with rTMS still remains unknown [21]. Further research is expected for the rTMS treatment and changes in the CBF in post-stroke aphasia. Our next challenge involves repeated high-frequency rTMS to right IFG in combination with iST to investigate the instant and long-term effects on the improvement of language function over time [10]. The compensation site associated with recovery of language function must, however, be studied using functional brain imaging, including fMRI, other than SPECT [6].

### Conclusions

The authors performed rTMS in combination with iST on a patient with post-stroke aphasia during hospitalization five times. rTMS was applied to right IFG at 1 Hz. His language function improved immediately, improved gradually over time, and stabilized in the latter stage. The CBF increased dominantly in right cerebral hemisphere. This article reports one case only, and the evaluation of CBF using the SPECT is also restricted. Further investigation is therefore required for the combined use of rTMS and iST on language function and CBF transition.

## Data Availability

The authors confirm that the data supporting the findings of this study are available within the article.

## Abbreviations

rTMS: Repetitive transcranial magnetic stimulation
iST: Intensive speech-language-hearing therapy
CBF: Cerebral blood flow
SPECT: Single photon emission computed tomography
FIM: Functional independence measure
ST: Speech-language-listening therapy
MRI: Magnetic resonance imaging
SLTA: Standard Language Test of Aphasia
RCPM: Raven’s coloured progressive matrices
IFG: Inferior frontal gyrus
SLTA-ST: Supplementary Tests for Standard Language Test of Aphasia
J-WAB: Japanese version of Western Aphasia Battery
J-Token Test: Japanese version of Token Test
rCBF: Regional cerebral blood flow (rCBF)
ECD: Ethyl cysteine dimer
3DSRT: Three-dimensional stereotactic regions of interest template
FineSRT: Fine stereotactic regions of interest template
ROI: Region of interest
fMRI: Functional MRI
